# Saucerneol D Suppresses the Growth of *Helicobacter pylori* and Their Virulence Factors

**DOI:** 10.3390/cimb47100828

**Published:** 2025-10-09

**Authors:** Su Man Kim, Hyun Jun Woo, Zhongduo Yang, Tiankun Zhao, Ji Yeong Yang, Sa-Hyun Kim

**Affiliations:** 1 Department of Clinical Laboratory Science, Semyung University, Jaecheon 27136, Republic of Korea; zeusuman@naver.com (S.M.K.); taesube@semyung.ac.kr (H.J.W.); 2 Department of Laboratory Medicine, Inje University College of Medicine, Busan 47392, Republic of Korea; 3 School of Life Science and Engineering, Lanzhou University of Technology, Lanzhou 730050, China; yangzhongduo@126.com (Z.Y.); zhaotiankun2006@163.com (T.Z.); 4 School of Mechanical Engineering, Yonsei University, Seoul 03722, Republic of Korea; 5 BK21 FOUR KNU Creative BioResearch Group, School of Life Sciences, Kyungpook National University, Daegu 41566, Republic of Korea

**Keywords:** *Helicobacter pylori*, saucerneol D, virulence factor

## Abstract

*Helicobacter pylori* infects the human stomach and causes various gastrointestinal diseases. Saucerneol D is a type of lignan, which is a polyphenol compound that exists naturally in plants, and it is abundant in flaxseed, sesame seeds, whole grains, vegetables, and fruits. Saucerneol D is found in *Saurus chinensis* extract and has been reported to exert a variety of effects, such as antioxidant and anti-inflammatory abilities. However, its antibacterial effect against *H. pylori* has not been reported; therefore, we analyzed the effect of saucerneol D on *H. pylori* in the present study. Changes in the expression of pathogenic factors and gene transcription in *H. pylori* were observed after treatment with saucerneol D using Western blotting and RT-PCR. It was confirmed that saucerneol D suppressed the growth of *H. pylori* by decreasing the expression of the genes *dna*N and *pol*A, which are required for bacterial replication. Saucerneol D also reduced the secretion of the major pathogenic toxin protein, CagA, by downregulating the expression of type IV secretion system-composing proteins. Furthermore, saucerneol D reduced ammonia production by inhibiting the expression of urease proteins, which are essential for the survival of *H. pylori* in the acidic gastric environment. Additionally, saucerneol D decreased the expression of *fla*B, potentially reducing motility. Finally, it was confirmed that the expression of the *sab*A gene, associated with cell adhesion, was reduced. These results suggest that saucerneol D inhibits the growth of *H. pylori* and the expression of several pathogenic factors, indicating that saucerneol D has an antimicrobial effect against *H. pylori*.

## 1. Introduction

*Helicobacter pylori* is a Gram-negative, spiral, and microaerophilic bacterium that is a significant pathogen responsible for various gastrointestinal diseases, including superficial gastritis, chronic gastritis, peptic ulcer, gastric mucosa-associated lymphoid tissue (MALT) lymphoma, intestinal metaplasia, and gastric cancer [1]. Thus, the World Health Organization classified *H. pylori* as a class I carcinogen in 1994 [2]. In recent years, the recommended primary treatment for *H. pylori* infection in Korea has been a triple-therapy regimen consisting of proton pump inhibitors (PPIs), clarithromycin, and amoxicillin for 10–14 days [3,4]. If the primary treatment fails, a secondary treatment using a four-drug regimen of PPIs, metronidazole, bismuth, and tetracycline can be employed [5]. Over the past decade, the rate of resistance to clarithromycin has increased, and the WHO has prioritized research and development of antibiotics to treat clarithromycin-resistant *H. pylori* [6,7]. Additionally, the tolerance rate to metronidazole has been reported to be over 15% worldwide [7]. These reports indicate that the antibiotic resistance rate of *H. pylori* is gradually increasing, necessitating the search for new treatments.

Cytotoxin-associated antigen A (CagA), the most important pathogenic factor in *H. pylori*, is encoded by the *cag* pathogenicity island (PAI) [8]. CagA is injected into gastric epithelial cells by the type IV secretion system (T4SS), which is also encoded by *cag*PAI [9]. It has been suggested that CagA significantly affects the transcriptional activation of interleukin-8 (IL-8) through the activation of mitogen-activated protein kinase (MAPK) and the nuclear factor kappa-light-chain-enhancer of activated B cells (NF-κB) and induces a cell membrane modification termed the hummingbird phenotype [10,11].

Another crucial *H. pylori* pathogenic factor, vacuolating cytotoxin A (VacA), is secreted by all *H. pylori* strains through the type Va secretion system (T5aSS) [12,13]. The secretion system subunit protein A (SecA) is an essential protein in T5aSS, providing the energy required for VacA to be secreted outside the bacterial membrane [14]. Once secreted, VacA binds to surface receptors on host cells to form anion channels, enters cells through clathrin-independent endocytosis, induces necrosis or apoptosis, and results in VacA-dependent vacuole formation [15,16].

Urease is an enzyme necessary for *H. pylori* to colonize the highly acidic environment of the stomach. Urease catalyzes the conversion of urea in the stomach into carbon dioxide (CO_2_) and ammonia (NH_3_). The ammonia produced neutralizes the gastric environment by increasing the pH [17]. Therefore, the urease enzyme is a known therapeutic target for the eradication of *H. pylori*.

**Figure 1:**
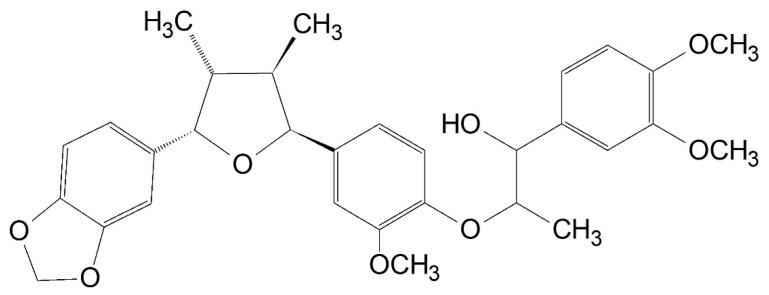
Chemical structure of saucerneol D.

*Saururus chinensis* (Lour.) Baill., a perennial herb native to East Asia, is distributed on Jeju island and mountain Jiri in Korea [18]. Extracts of *S. chinensis* have been studied for their antioxidant activity, hepatocellular protection, and antibacterial properties. Saucerneol D, a lignan found in the roots of *Saururus chinensis*, has been used to treat several inflammatory conditions, including jaundice, pneumonia, edema, and fever [18,19]. Saucerneol D, one of the lignans of *S. chinensis*, has the chemical structure shown in Figure 1 [20]. However, there is no report of saucerneol D having antibacterial effects. Therefore, this research investigated the inhibitory effect and mechanism of saucerneol D on *H. pylori*, as well as its impact on the reduction in various virulence factors of *H. pylori* in vitro.

## 2. Materials and Methods

### 2.1. Bacterial Culture

The *H. pylori* reference strain of ATCC 49503 was purchased from the American Type Culture Collection (ATCC, Manassas, VA, USA). *H. pylori* were grown on Brucella agar plates (BD Biosciences, Franklin Lakes, NJ, USA), with selectively supplemented *H. pylori* (Oxoid Limited, Hampshire, UK) and heat-inactivated 10% bovine serum (Gibco, Grand Island, NE, USA) placed under microlender microaerophilic and 100% humidity conditions at 37 °C and inspected after three to four days.

### 2.2. Determination of Minimum Inhibitory Concentration (MIC)

For the dilution test, *H. pylori* ATCC 49503 strain colonies grown on Brucella agar plates were collected and resuspended in sterile saline. Saucerceol D was obtained from the Natural Products Bank, National Institute for Korean Medicine Development (Gyeongsan, Gyeongsangbuk, Republic of Korea), and was dissolved using DMSO (dimethyl sulfoxide) as a solvent. To test the agar dilution, the number of bacterial particles in the *H. pylori* suspension was set to McFarland 3.3. An amount of 10 µL of the bacterial suspension was placed on Mueller–Hinton agar plates with heat-inactivated 10% bovine serum including indicated concentrations of saucerneol D. For liquid dilution experiments, the number of bacterial particles in the *H. pylori* suspension was set to a McFarland scale of 0.5. Bacteria were cultured for 72 h in broths treated with various concentrations of saucerneol D. All the solutions were prepared in such a manner that the final DMSO concentration was the same in all treatments. The final optical density (600 nm) of the bacterial suspension was measured by spectrophotometry.

### 2.3. RNA Extraction and Reverse Transcriptase Polymerase Chain Reaction (RT-PCR)

Cultured *H. pylori* were washed twice with sterile saline, and total RNA was extracted using easy-BLUE™ Total RNA Extraction Kit (iNtRON Biotechnology, Seongnam, Republic of Korea) as described in the manufacturer’s instructions. The reverse transcriptase polymerase chain reaction (RT-PCR) primer sequences used in this study are listed in Table 1. *Gal*E (UDP-Galactose-4-Epimerase) was used as an internal control. Gel images were taken using the Molecular Image Gel DocTM XR+ system (Bio-Rad, Hercules, CA, USA). The band intensity of PCR product was analyzed with the ImageLab 6.1 software (BioRad, Hercules, CA, USA).

### 2.4. Anti-H. pylori SecA or Anti-H. pylori Polyclonal Antibody Production

We produced rabbit anti-SecA or rabbit anti-*H. pylori* (ATCC 49503) polyclonal antibodies following a method described previously [25]. New Zealand White rabbits (8~10 weeks) were purchased from Central Lab Animal Inc. (Seoul, Republic of Korea), and the SecA polyclonal antiserum was produced by injection of 500 μg of 22 mer peptides (SecA: GTERHESRRIDNQLRGRSGRQG; nucleotide 518–539) through ear vein. The *H. pylori* polyclonal antibodies were generated by immunization with intravenous injection with formaldehyde-fixed 1 × 10^8^/mL concentration of *H. pylori* (ATCC 49503) bacteria every week for a total of six weeks. After six weeks, about 100 mL of whole blood was collected from the heart and then allowed to clot in a refrigerator overnight, and the serum was isolated after centrifugation. Pre-immune serum was harvested prior to immunization. Antibodies were purified using a protein A column. The rabbit anti-SecA antibodies were used at a 1:5000 dilution in 5% skim milk blocking solution. The rabbit anti-*H. pylori* (ATCC 49503) polyclonal antibodies were used at a 1:10,000 dilution.

### 2.5. Protein Extraction and Western Blot

The bacterial cell lysate was prepared to detect SecA molecules. Bacteria were lysed with a radio immunoprecipitation assay lysis buffer (Biosesang, Seongnam, Republic of Korea) containing a protease inhibitor cocktail, it was then incubated on ice for 30 min. To lyse the bacterial cells completely, the mixture was sonicated for 2 min in 10 s intervals (Sonicator XL-2020, Heat Systems Ultrasonics, Pittsburgh, PA, USA). The cell lysates were then centrifuged at 14,000 rpm at 4 °C for 10 min, and the supernatants were collected. Proteins were quantified by Lowry Protein assay (Bio-Rad) and mixed with ×5 sodium dodecyl sulfate (SDS) sample loading buffer. Protein samples were separated by SDS-polyacrylamide gel electrophoresis for 90 min at 120 voltage and transferred to a nitrocellulose membrane for 90 min at 400 mA. The bacterial culture supernatant was also collected to detect CagA VacA or urease, and then it was concentrated 10-fold using Amicon Centricon centrifugal filters (3 kDa cut-off) (Millipore, Billerica, MA, USA) at 3000 rpm for 2 h at 4 °C. Polyclonal antibodies against whole *H. pylori* ATCC 49503 proteins were used as an internal control.

### 2.6. Urease Activity Test

*H. pylori* ATCC 49503 strain was grown in urea broth containing 0.1% urea with the indicated concentrations of saucerneol D (12.5, 25, and 50 ng/mL) and acetohydroxamic acid (AHA) (50 ng/mL). After 1 h, centrifugation was performed at 3000 rpm for 10 min and then 1 mL of supernatant was extracted. Urease activity was confirmed by measuring the amount of ammonia using an Asan Set Ammonia kit (Asan Pharmaceutical, Seoul, Republic of Korea) according to the manufacturer’s instructions. Ammonia concentrations in the specimens were calculated using the standard curve.

### 2.7. Statistical Analysis

Data in the bar graphs are presented as mean ± standard error of mean (SEM). All the statistical analyses were performed using GraphPad Prism 7.0 software (GraphPad Software, San Diego, CA, USA). All the data were analyzed using an unpaired Student’s *t*-test, and *p* < 0.05 was considered statistically significant (* *p* < 0.05, ** *p* < 0.01, and *** *p* < 0.001). Every experiment was repeated at least three times to verify the results and perform statistical analysis.

## 3. Results

### 3.1. Determination of the MIC

**Figure 2:**
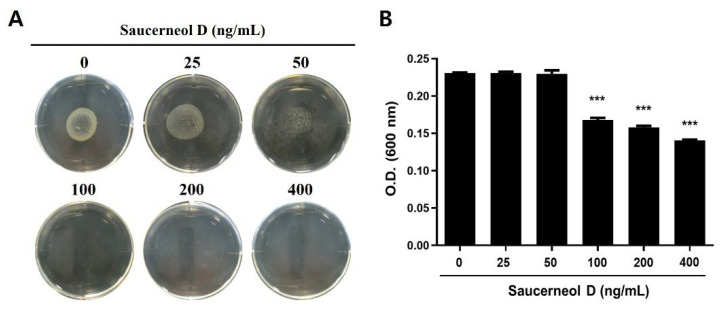
Determination of the MIC of saucerneol D against *H. pylori*. The MIC of saucerneol D (25, 50, 100, 200, and 400 ng/mL) was measured for *H. pylori* reference strain ATCC 49503 using (**A**) the agar dilution method and (**B**) the broth dilution method. The results from three independent experiments were analyzed using Student’s *t*-test (*** *p* < 0.001).

The agar dilution test was performed to determine the minimum inhibitory concentration (MIC) of saucerneol D against *H. pylori*. Mueller–Hinton agar containing heat-inactivated 10% bovine serum and varying concentrations of saucerneol D (25, 50, 100, 200, and 400 ng/mL) was prepared, and *H. pylori* were grown on the agar plates for 72 h. According to the agar dilution test, the MIC of saucerneol D against *H. pylori* was found to be 100 ng/mL, indicating that even low concentrations were sufficient to suppress bacterial growth (Figure 2A). The MIC was also confirmed by a broth dilution test, which showed bacterial growth inhibition at concentrations above 100 ng/mL of saucerneol D, consistent with the agar dilution test results (Figure 2B). Therefore, to avoid bactericidal conditions and better assess mechanistic effects, subsequent experiments were performed with saucerneol D concentrations below 50 ng/mL.

### 3.2. Effect on Replication and Transcription Genes in Saucerneol D-Treated H. pylori

**Figure 3:**
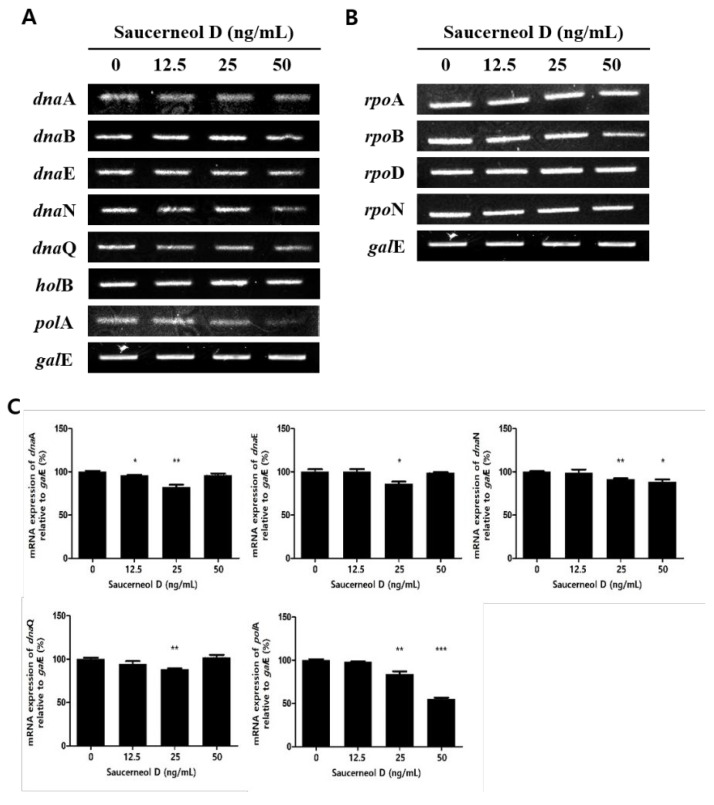
Effect on replication and transcription genes of *H. pylori*. The collected RNA was subjected to RT-PCR to detect the mRNA expression levels in saucerneol D-treated *H. pylori*. (**A**) The replication genes: *dna*A, *dna*B, *dna*E, *dna*N, *dna*Q, *hol*B, and *pol*A. (**B**) The transcription genes: *rpo*A, *rpo*B, *rpo*D, and *rpo*N. (**C**) Each band intensity was normalized to *gal*E. Data are presented as mean ± SEM. The results from three independent experiments were analyzed using Student’s *t*-test (* *p* < 0.05, ** *p* < 0.01, and *** *p* < 0.001).

To investigate the mechanism behind the inhibitory effect of saucerneol D on *H. pylori*, the bacteria were treated with sub-MICs of saucerneol D (12.5, 25, and 50 ng/mL). The results showed that the mRNA expression levels of *dna*A, *dna*N, *dna*Q, and *pol*A, among the replication genes of *H. pylori*, were decreased in saucerneol D-treated *H. pylori* (Figure 3A,C). In contrast, saucerneol D did not affect the transcription genes *rpo*A, *rpo*B, *rpo*D, and *rpo*N (Figure 3B,C).

### 3.3. Effect on CagA, VacA, and Their Secretion Systems in Saucerneol D-Treated H. pylori

**Figure 4:**
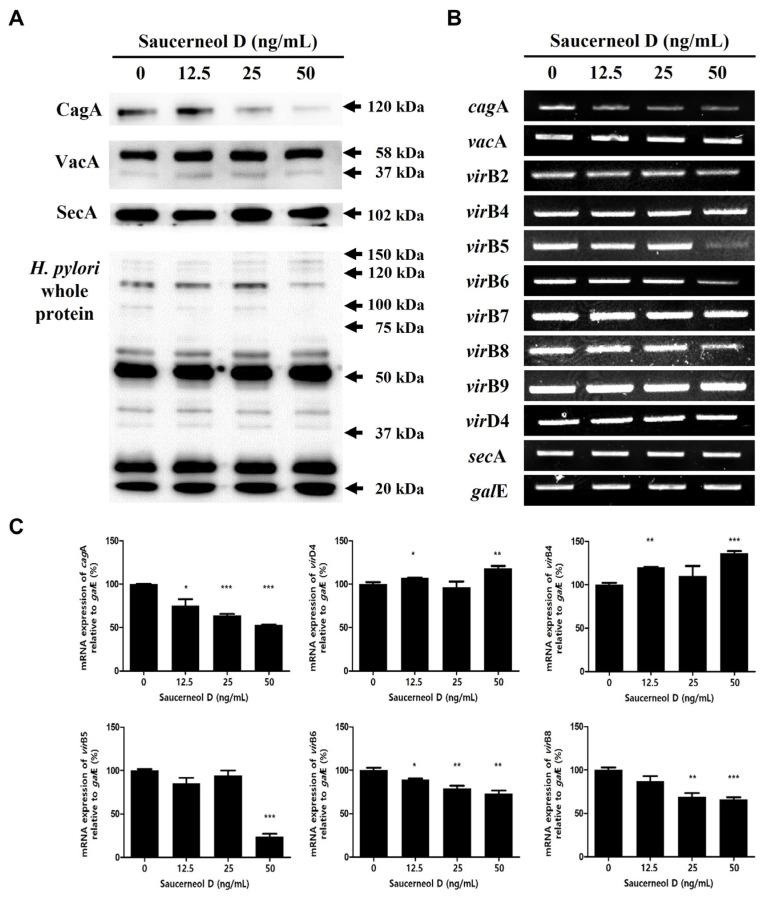
Effects on CagA, VacA, and each secretion system in saucerneol D-treated *H. pylori*. (**A**) The culture supernatant and bacterial cell lysates were subjected to Western blotting to detect supernatant CagA, VacA, or intracellular SecA. The rabbit anti-*H. pylori* polyclonal antibody was used as an internal control. (**B**) The collected RNA was subjected to RT-PCR to detect the mRNA expression levels of *cag*A, *vac*A, T4SS genes (*vir*B2, *vir*B4, *vir*B5, *vir*B6, *vir*B7, *vir*B8, and *vir*D4), and *sec*A. (**C**) Each PCR band intensity was normalized to *gal*E. Data are presented as mean ± SEM. The results from three independent experiments were analyzed using Student’s *t*-test (* *p* < 0.05, ** *p* < 0.01, and *** *p* < 0.001).

*H. pylori* were treated with saucerneol D (12.5, 25, and 50 ng/mL) for 72 h in Mueller–Hinton broth containing heat-inactivated 10% bovine serum. After harvesting the protein and mRNA, Western blotting and RT-PCR analyses were performed. Saucerneol D decreased *cag*A expression and CagA protein secretion in a concentration-dependent manner, but the mRNA and protein secretions of VacA were not affected by saucerneol D (Figure 4A–C). Among the components of T4SS, which is the CagA secretory pathway, the expressions of *vir*B5, *vir*B6, and *virB8* mRNA were decreased by saucerneol D, whereas those of *vir*B4 and *vir*D4 were increased somewhat (Figure 4B,C). The mRNA and protein expressions of SecA, a regulating component of the T5aSS for VacA secretion, were not affected by saucerneol D (Figure 4A–C).

### 3.4. Downregulation of Urease in Saucerneol D-Treated H. pylori

**Figure 5:**
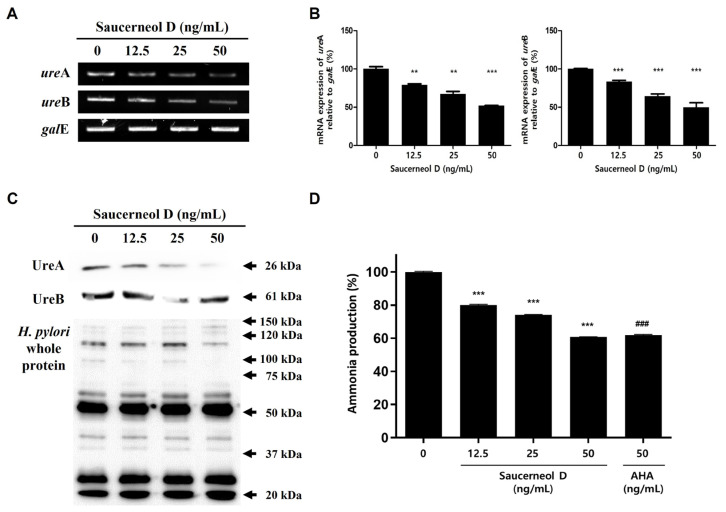
Effects on urease expression or secretion in saucerneol D-treated *H. pylori.* (**A**) The collected RNA was subjected to RT-PCR testing to detect the mRNA expression levels of urease subunits (*ure*A and *ure*B). (**B**) Each PCR band intensity was normalized to *gal*E. (**C**) The bacteria lysates were subjected to Western blotting to detect UreA and UreB. The rabbit anti-*H. pylori* polyclonal antibody was used as an internal control. (**D**) *H. pylori* were exposed to indicated concentrations of saucerneol D (12.5, 25, and 50 ng/mL) and AHA (50 ng/mL) for 72 h. The collected supernatant measured the amount of ammonia, and the results were compared with the non-treated group. Data are presented as mean ± SEM. The results from three independent experiments were analyzed using Student’s *t*-test (** *p* < 0.05, *** *p* < 0.001, and ^###^ *p* < 0.001).

*H. pylori*’s urease enzyme is vital for its survival and colonization of the gastric mucosa [12]. It is composed of two subunits, UreA and UreB, encoded by *ure*A and *ure*B, respectively [26]; both of these were inhibited after treatment with saucerneol D (Figure 5A–C). To confirm the effect of reduced UreA and UreB expression on urease activity, the amount of ammonia produced by urease was measured. *H. pylori* were grown in brucella broth containing 0.1% urea with the indicated concentrations of saucerneol D (12.5, 25, and 50 ng/mL) and AHA (50 ng/mL). After 1 h of incubation, the ammonia produced was measured. Ammonia levels decreased in a concentration-dependent manner after treatment with saucerneol D, reaching similar levels as those treated with AHA at the same concentration (Figure 5D).

### 3.5. Effect on Flagella and Adhesion in Saucerneol D-Treated H. pylori

**Figure 6:**
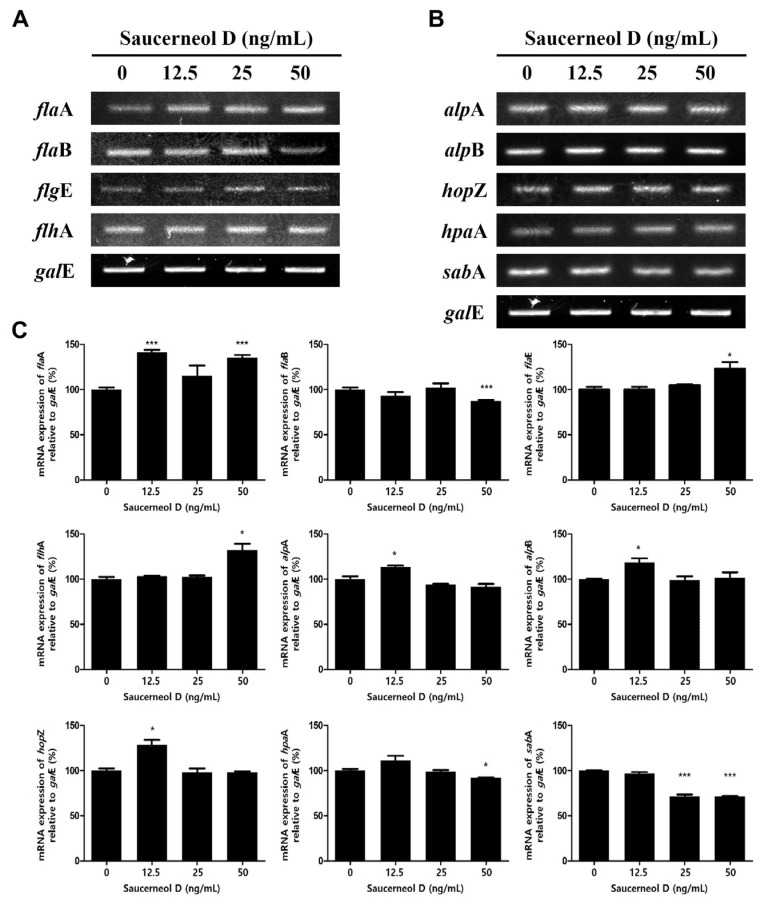
Effect on the expression of flagella and adhesion genes in saucerneol D-treated *H. pylori.* The collected RNA was subjected to RT-PCR to detect the mRNA expression levels of (**A**) flagella genes (*fla*A, *fla*B, *fla*E, and *flh*A) and (**B**) adhesion genes (*alp*A, *alp*B, *hop*Z, *hpa*A, and *sab*A). (**C**) Each PCR band intensity was normalized to *gal*E. Data are presented as mean ± SEM. The results from three independent experiments were analyzed using Student’s *t*-test. (* *p* < 0.05 and *** *p* < 0.001).

The movement and adhesion of *H. pylori* are critical for the successful infection of gastric epithelial cells. *H. pylori*’s movement is facilitated by flagella [27]. This study aimed to determine the effects of saucerneol D on the expression of flagella genes (*fla*A*, fla*B*, flg*E*,* and *flh*A) and adhesion genes (*alp*A*, alp*B*, hop*Z*, hpa*A*,* and *sab*A). Saucerneol D increased the expression of *fla*A*, flg*E*,* and *flh*A among the flagella genes while decreasing the expression of *fla*B (Figure 6A,C). These findings suggest that saucerneol D may interfere with the balance of flagellar components, potentially altering motility. Additionally, among the adhesion genes, only the expression of *sab*A was decreased by saucerneol D (Figure 6B,C). Together, these results indicate that saucerneol D not only affects bacterial motility genes but may also weaken the adhesion capacity of *H. pylori*.

## 4. Discussion

*H. pylori* is a major cause of gastric cancer, accounting for 75% of all cases [28,29]. However, the increasing resistance to clarithromycin and the limitations of current primary therapies have been widely reported [6,30,31]. This highlights the need for research into new therapeutic agents or adjuvants to inhibit *H. pylori*. Although the antibacterial activity of saucerneol D, which is a lignan, has not been studied, the antibacterial activity of extracts from *Saururus chinensis* (Lour.) Baill. has been documented [18,19]. This study investigated the inhibitory effects of saucerneol D, a component of the extract, on *H. pylori* and its pathogenic factors.

**Figure 7:**
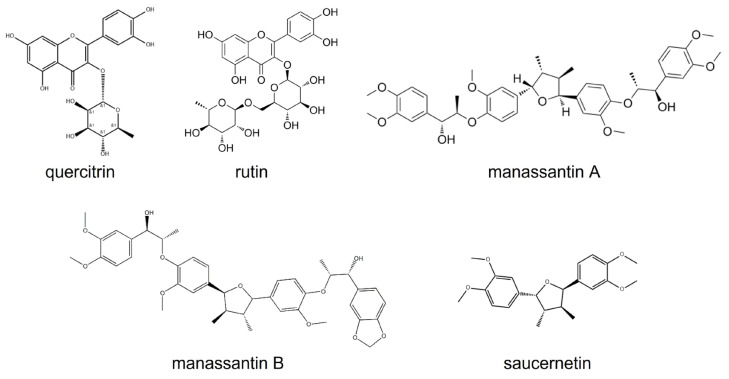
Extracts from *S. chinensis* (Lour.) Baill. contain quercetin glycosides such as quercitrin and rutin and lignans such as manassantin A, manassantin B, and saucernetin, including saucerneol D.

Extracts from *S. chinensis* (Lour.) Baill. are known to contain quercetin glycosides such as quercitrin and rutin, as well as lignans such as saucerneol D, manassantin A, manassantin B, and saucernetin, and their structural formulas are displayed in Figure 7 [20]. It appears that, along with other constituents of the crude extract, saucerneol D contributes to anti-*H. pylori* activity. Saucerneol D inhibited *H. pylori* growth by downregulating essential replication machinery genes (Figure 2 and Figure 3). Specifically, it reduced the mRNA expression of *dna*N and *pol*A (Figure 3). DNA polymerase III, which is crucial for bacterial replication, comprises core polymerases (DnaE, DnaQ, and HolE), a sliding clamp (DnaN), and a multiprotein clamp loader (DnaX, HolA, HolB, HolC, and HolD) [32]. DNA polymerase I, encoded by the *pol*A gene, plays a key role in connecting Okazaki fragments, repairing DNA, and replacing RNA primers with DNA [33]. Since DnaN and PolA are indispensable for bacterial replication, this result suggests that saucerneol D inhibits *H. pylori* growth by targeting these replication components.

Saucerneol D not only inhibited the synthesis of CagA, a critical virulence factor of *H. pylori*, but it also increased the mRNA expression of *vir*B4, *vir*B9, and *vir*D4 of the T4SS, which injects CagA into host cells, while decreasing *vir*B5, *vir*B6, and *vir*B8 (Figure 4). VirB4 and VirD4 are required for substrate recruitment and transport to T4SS, VirB6 stabilizes VirB5, and VirB8 is involved in assembling the inner membrane complex [9,34,35]. VirB9 forms a transmembrane pore complex, essential for transferring the matrix through the peri-membrane space and anchoring to the membrane [36]. VirB5 links external pilus recipients and donor cells and is a component of the T4SS external pilus [9,37]. Overall, the reduction in pathogenic factors and key secretory pathway components suggests that CagA translocation into host cells might be hindered. The preferential effect on CagA–T4SS over VacA pathways suggests possible selectivity, which warrants further mechanistic investigation.

Urease secretion is also regulated by T5aSS [25]. SecA, an important protein constituting the T5aSS, provides the energy for urease or VacA secretion outside the cell membrane [14,25,38]. Although there were no changes in the transcription of *sec*A or expression of SecA after saucerneol D treatment (Figure 4A,B), the reason for the decrease in urease secretion is thought to be due to the decrease in the expression of *ure*A and *ure*B (Figure 5A). Urease-negative mutant *H. pylori* cannot colonize the stomachs of nude mice or gnotobiotic piglets, indicating that urease activity is essential for colonization [39,40]. If saucerneol D caused a reduction in urease secretion, as suggested in Figure 5, this might deteriorate *H. pylori*’s colonization and viability in the acidic environment of the stomach. The decrease in UreA and UreB synthesis led to reduced ammonia production (Figure 5D). Ammonia not only neutralizes stomach acid but also directly damages gastric epithelial cells and induces inflammation [41,42]. Additionally, the CO_2_ produced by urease protects *H. pylori* from oxidative damage by inhibiting peroxynitrite, thus enhancing viability [43,44]. Overall, saucerneol D might reduce urease synthesis in *H. pylori*, preventing gastric environment neutralization, decreasing colonization and survival rates, and potentially reducing the inflammatory response of gastric epithelial cells.

Saucerneol D also downregulated *fla*B but upregulated *fla*A, *flg*E, and *flh*A among the *H. pylori* flagella genes (Figure 6A). FlaA and FlaB are flagellins, making up the flagellar filament [44,45]. FlgE anchors flagellar filaments to the export domain of the basal body, while FlhA is part of the export dome and regulates flagellin protein release [46,47]. Although *fla*B decreased, the increase in the other flagella genes suggests that *H. pylori*, when exposed to saucerneol D, might have a compensatory mechanism to escape saucerneol D stress.

Saucerneol D also suppressed the expression of *sab*A, an adhesion gene in *H. pylori* (Figure 6B). *H. pylori* adhesion is a key mechanism in inducing chronic gastritis and gastric cancer, with chronic infection increasing inflammation and sialyl-Lewis X expression [48,49,50]. SabA mediates *H. pylori* binding to inflamed gastric mucosa by recognizing sialyl-Lewis A and sialyl-Lewis X antigens [51]. Thus, the inhibition of *sab*A expression by saucerneol D may reduce *H. pylori* adhesion, chronic infection, and the inflammatory response.

Further experiments using human gastrointestinal cell models are needed to confirm the effects of saucerneol D on CagA and VacA translocation, host cell adherence, and motility. Moreover, in vivo experiments using animal infection models are needed to substantiate these findings, and the bioavailability and gastric stability of saucerneol D remain unknown. Future studies should evaluate these properties in simulated gastric fluids and in vivo models. In addition, the potential toxicity and selectivity of saucerneol D toward host cells should be evaluated in future studies to assess its suitability as a therapeutic candidate.

This study demonstrated that saucerneol D inhibits the growth of *H. pylori* and reduces its virulence factors. To summarize, saucerneol D decreased the expression of CagA virulence factors and related secretion systems, including T4SS. Saucerneol D also downregulated urease subunit proteins, thereby reducing urease activity.

## 5. Conclusions

**Figure 8:**
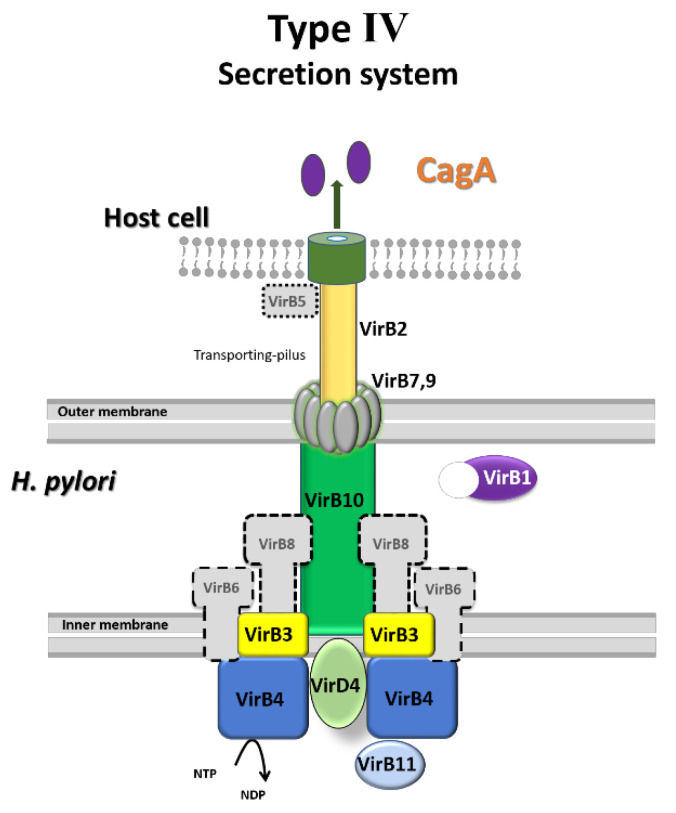
Inhibitory effect on the expression of VirB5, VirB6, and VirB8, which comprise the T4SS components in saucerneol D-treated *H. pylori*.

In conclusion, we found that saucerneol D acts as a potent inhibitor of *H. pylori* multiplication and of virulence factors—CagA, urease, and *sab*A—involved in *H. pylori* pathogenesis. *H. pylori* growth was inhibited through the downregulation of its replication machinery when exposed to saucerneol D (Figure 3). In addition, saucerneol D decreased the CagA secretion of *H. pylori* by downregulating the T4SS-comprising molecules VirB5, VirB6, and VirB8 (Figure 8). Saucerneol D also downregulated the expression of *H. pylori* urease subunit proteins, thereby reducing urease activity. The expression of an adhesion molecule gene, *sab*A, was also reduced when exposed to saucerneol D. Following these results, we suggest saucerneol D as a potential *H. pylori* inhibitor. We have plans to re-examine the anti-*H. pylori* effect of saucerneol D in the future using a gastric cell line infection model or a Mongolian gerbil infection model.

## Figures and Tables

**Table 1:** List of primer sequences and PCR conditions for RT-PCR.

Primers	Sequences (5′-3′)		Product Length (bp)	Annealing Temperature (°C)	Cycles	Reference
	Forward	Reverse				
*dna*A	GGGCATGACTTTAGCGGTTA	TTAACGAATTGCACGCCAAC	128	55	24	[21]
*dna*B	AATGGGCCGTTTATCGTCTC	CAAATCCGCTTGCAACTACG	231	55	30	
*dna*E	AATCCACCGGCTCCAAATAC	GCCAAACAAGTGTGGGAGTA	184	55	30	
*dna*N	GTTAGCGGTGGTTGAAAACG	CGGTTTCGCTATGCTCAGAA	233	55	30	
*dna*Q	CGCATGAAGCTTTGCAAGAA	GCATAGGCTCTATGGCTGAC	244	55	30	
*hol*B	TGCAAGCCTTTTTGAACACC	CGCGTTTTGGGCTTCTATAC	196	55	30	
*pol*A	TTTCCAAGCTCCCTAATCGC	ATACGGGATTTTGCCGAGTC	134	55	30	[22]
*rpo*A	AGCGACACGTCTTCAGTAAC	ACAGCACCTTTGATCCCATC	224	55	30	[21]
*rpo*B	TTTAGGTAAGCGCGTGGATT	AATCAGCTTTGGATGGAACG	301	59	24	
*rpo*D	TCATCATCATTGCCGACTGG	GTCATGCGCAAACACATTCA	152	55	27	
*rpo*N	GCCCTTGAAATCGTGCTTAC	ATGATGAGAGCTACCCGACA	250	55	27	
cagA	GTCATAATGGCATAGAACCTGAA	ATTCCCTAGGGCGTCTAAATAA	407	59	27	[23]
*vac*A	AAACGACAAGAAAGAGATCAGT	CCAGCAAAAGGCCCATCAA	291	57	22	
*sec*A	AAAAATTTGACGCTGTGATCC	CCCCCAAGCTCCTTAATTTC	274	47	27	
*ure*A	GCCAATGGTAAATTAGTT	CTCCTTAATTGTTTTTAC	411	40	20	[24]
*ure*B	CCATCCACGAACACATGGTA	TCTATCCCTACCCCACAACC	252	50	20	
*vir*B2	CAGTCGCCTGACCTCTTTTGA	CGGTCACCAGTCCTGCAAC	156	62	30	
*vir*B4	GTTATAGGGGCAACCGGAAG	TTGAACGCGTCATTCAAAGC	449	62	30	
*vir*B5	TACAAGCGTCTGTGAAGCAG	GACCAACCAACAAGTGCTCA	436	62	36	
*vir*B6	CCTCAACACCGCCTTTGGTA	TAGCCGCTAGCAATCTGGTG	225	62	30	
*vir*B7	GATTACGCTCATAGGCGATGC	TGGCTGACTTCCTTGCAACA	202	62	30	
*vir*B8	GTTGATCCTTGCGATCCCTCA	CGCCGCTGTAACGAGTATTG	218	62	25	
*vir*B9	GCATGTCCTCTAGTCGTTCCA	TATCGTAGATGCGCCTGACC	269	62	34	
*vir*D4	CCGCAAGTTTCCATAGTGTC	GCGAGTTGGGAAACTGAAGA	263	62	30	
*flh*A	TCATTGGAGGGTTTTTAGTGG	GGTGCGAGTGGCGACAAT	155	60	35	
*fla*A	TAGACACCACCAACGCTAAA	TGCATTCTAGGGGGTTGTAT	239	62	32	
*fla*B	GTCAATGGCGTGAATGATTA	ATTCACGGTCCCAATTTCTA	213	52	32	
*flg*E	CCGATCAAATCCTTAACACC	AGGCTTAAAAACATGCGAAC	381	52	30	
*sab*A	AAAGCATTCAAAACGCCAAC	CCCGCATAAAGACTCCAAAA	163	60	26	
*hop*Z	GCGCCGTTACTAGCATGATCA	GAAATCTTTCGGCGCGTTT	101	60	26	
*hpa*A	GAGCGTGGTGGCTTTGTTAGT	TCGCTAGCTGGATGGTAATTCA	90	60	26	
*alp*A	GCACGATCGGTAGCCAGACT	ACACATTCCCCGCATTCAAG	90	60	27	
*alp*B	ACGCTAAGAAACAGCCCTCAAC	TCATGCGTAACCCCACATCA	82	60	28	

## Data Availability

The original contributions presented in this study are included in the article material. Further inquiries can be directed to the corresponding author(s).
